# The MADS-Box transcription factor Bcmads1 is required for growth, sclerotia production and pathogenicity of *Botrytis cinerea*

**DOI:** 10.1038/srep33901

**Published:** 2016-09-23

**Authors:** Zhanquan Zhang, Hua Li, Guozheng Qin, Chang He, Boqiang Li, Shiping Tian

**Affiliations:** 1 Key Laboratory of Plant Resources, Institute of Botany, Chinese Academy of Sciences, Beijing, China

## Abstract

MADS-box transcription factors are highly conserved in eukaryotic species and involved in a variety of biological processes. Little is known, however, regarding the function of MADS-box genes in *Botrytis cinerea*, a fungal pathogen with a wide host range. Here, the functional role of the *B. cinerea* MADS-box gene, *Bcmads1,* was characterized in relation to the development, pathogenicity and production of sclerotia. The latter are formed upon incubation in darkness and serve as survival structures during winter and as the female parent in sexual reproduction. *Bcmads1* is indispensable for sclerotia production. RT-qPCR analysis suggested that *Bcmads1* modulated sclerotia formation by regulating the expression of light-responsive genes. *Bcmads1* is required for the full virulence potential of *B. cinerea* on apple fruit. A comparative proteomic analysis identified 63 proteins, representing 55 individual genes that are potential targets of *Bcmads1*. Among them, Bcsec14 and Bcsec31 are associated with vesicle transport. Deletion of *Bcsec14* and *Bcsec31* resulted in a reduction in the virulence and protein secretion of *B. cinerea*. These results suggest that *Bcmads1* may influence sclerotia formation by modulating light responsive gene expression and regulate pathogenicity by its effect on the protein secretion process.

*Botrytis cinerea* is a necrotrophic pathogen and the causal agent of grey mold disease in more than 200 crop species, including almost all vegetable and fruit crops^1^. Grey mold brings astonishing economic losses each year worldwide and the annual global costs of *Botrytis* control easily surmount € 1 billion^2^. Due to its importance in agricultural production and the available genomic resources, *B. cinerea* has become a useful model for studying the pathogenesis of aggressive fungal pathogens. To date, however, the development of new strategies for battling grey mold disease has been challenged by a lack of deep understanding of the pathogenesis of *B. cinerea*. The pathogenicity of plant pathogens can be affected by many factors, such as signal transduction components^3^,^4^, ROS generating systems^5^,^6^, and extracellular enzymes^7^,^8^,^9^,^10^. The regulation of pathogenicity by transcription factors has received considerable attention in recent studies of plant pathogens. Transcription factors play a central role in the regulation of gene expression at the transcriptional level. Individual transcription factors bind directly to specific motifs in the promoter region of genes, thereby modulating gene expression and regulating downstream biological processes.

The MADS-box family transcription factors are highly conserved in eukaryotic organisms and are involved in the regulation of many different cellular functions^11^,^12^. In fungi, MADS-box proteins can be classified into two types, SRF-type (type I) and MEF-type (type II). *Saccharomyces cerevisiae* contains four MADS-box transcription factors; Mcm1 and Arg80 belong to the SRF-type, and Rlm1 and Smp1 belong to the MEF-type. Rlm1 regulates the expression of genes required for cell wall integrity, while Smp1 is involved in osmotic stress response^13^,^14^,^15^. Mcm1 and Arg80 participate in the regulation of arginine metabolism in yeast cells^16^,^17^. The genome of the hemibiotrophic plant pathogen *Magnaporthe oryzae* contains two genes encoding putative MADS-box transcription factors, *Mig1* and *MoMcm1*. Mig1, an orthologue of *S. cerevisiae* Rlm1, is important for pathogenesis, although it is not required for appressorium formation^18^. Results of that study indicated that Mig1 is a downstream target of the Mps1 MAP kinase. The other MADS-box transcription factor MoMcm1 in *M. oryzae* is the orthologue of Mcm1 in *S. cerevisiae* and is required for male fertility, microconidium production, and virulence^19^. MoMcm1 interacts with Mst12 and MatA-1 proteins that regulate germ tube identity and male fertility, respectively^19^. The Mcm1 orthologue in the basidiomycete *Ustilago maydis* plays an important role in controlling the mating efficiency and expression level of pheromone-inducible genes^20^. Recently, the *Fusarium graminearum* MADS-box transcription factor Fgmcm1 was confirmed to play a role in the regulation of secondary metabolism, conidiogenesis, sexual development, cell identity, and virulence^21^. In the necrotrophic pathogen *Sclerotinia sclerotiorum*, the function of the MADS-box transcription factor gene *SsMADS* was investigated using RNA interference^22^. Results indicated that when the expression of *SsMADS* was silenced, the growth rate and virulence of *S. sclerotiorum* were significantly repressed. Although the function of MADS-box transcription factors in the virulence of various fungal pathogens has been demonstrated, little is known about their targets.

In the present study, the function of the *B. cinerea* MEF-type MADS-box transcription factor *Bcmads1* was investigated. Results indicated that *Bcmads1* is involved in the regulation of growth, sclerotia formation, and pathogenesis. A comparative proteomic study was conducted in order to elucidate the regulatory mechanisms associated with *Bcmads1.* Results indicated that *Bcmads1* regulates the abundance of Bcsec14 and Bcsec31, which are proteins that are associated with protein secretion. Deletion of *Bcsec14* or *Bcsec31* impaired the virulence of *B. cinerea* on apple fruits and tomato leaves. Collectively, these findings suggest that *Bcmads1* may affect the pathogenicity of *B. cinerea* by influencing protein secretion.

## Materials and Methods

### Strains and culture conditions

*B. cinerea* strain B05.10 was used as the recipient strain for gene replacement and complementation, as well as the wild-type strain in all experiments. The wild-type and genetically engineered strains of *B. cinerea* were cultured on potato dextrose agar (PDA) at 22 °C. Potato dextrose broth (PDB) and Czapeck liquid medium (2.5 g NaNO_3 _L^−1^, 0.5 g KCl L^−1^, 0.5 g MgSO_4_·7H_2_O L^−1^, 10 mg FeSO_4_·7H_2_O L^−1^, 1 g K_2_HPO_4 _L^−1^, 10 g apple pectin L^−1^) were used to culture *B. cinerea* for the extraction of intracellular or extracellular proteins.

### Construction of the *Bcmads1* deletion and complementation vectors

Primers for amplifying *Bcmads1* were designed based on the *B. cinerea* genome sequence, in which the *Bcmads1* gene ID is Bcin05g04030. To construct the knockout vector of *Bcmads1*, the upstream flank L (1195 bp) and downstream flank R (1150 bp) regions of the *Bcmads1* gene were amplified from *B. cinerea* genomic DNA by PCR using the primer pairs ML-F/ML-R and MR-F/MR-R, respectively. Restriction sites were added at both ends of flank L and flank R as shown in Supplementary Fig. S1A. Flank L and flank R were ligated into the corresponding site of the pLOB7 vector (provided by Jan van Kan, Wageningen Agricultural University, The Netherlands), which carries the hygromycin phosphotransferase gene *hph* under control of the *Aspergillus nidulans oliC* promoter and *trpC* terminator, which confers resistance to hygromycin. The fragment used for transformation was amplified from the constructed vector using the primer pair ML-F/MR-R.

The complementation vector was generated using the yeast gap repair approach^23^. *Bcmads1* including 1,000 bp of the promoter region was amplified using the primer pair B-C-F/B-C-R and co-transformed into *S. cerevisiae* FY834 with the SpeI/NotI-digested plasmid pNAN-OGG (provided by Julia Schumacher, Westfaelische Wilhelms-Universitaet Muenster, Germany), the latter of which confers resistance to nourseothricin, resulting in pBcmads1-C. The fragment used for creating the complementation strain from the *Bcmads1* deletion mutant was amplified using the primer pair A5-F/A3-R from the constructed vector pBcmads1-C. The vector pNAN-OGG contains gene flanks of *bcniiA* (nitrite reductase) of *B. cinerea,* allowing for targeted integration at the respective gene locus. Before transformation, the complementary fragment was completely sequenced in order to ensure complete fidelity with the original sequence.

### Transformation of *B. cinerea* and diagnostic PCR to detect homologous recombination

Protoplast formation and transformation of *B. cinerea* were performed using the methods described by Zhang *et al*.^24^. The replacement cassette used for protoplast transformation was amplified using the primer pair ML-F/MR-R and 100 μg of the PCR product was used for each transformation. Hygromycin resistant transformants were analyzed by PCR using the primer pair MJ-F/MJ-R. The sense primer MJ-F is located upstream of flank L and the antisense primer MJ-R is located inside the *oliC* promoter of the hygromycin resistant cassette. In order to obtain homokaryotic strains, positive transformants were purified by single-spore isolation. The homokaryotic transformants were then subjected to Southern blot analysis in order to exclude ectopic integration.

### Construction of the *Bcmads1-GFP* fusion vector and fluorescence microscopy

The *Bcmads1-GFP* fusion vector was generated using the yeast gap repair method^23^. The open reading frame of *Bcmads1* was amplified using the primer pair M-N-GFP-F/M-N-GFP-R, fused with the NotI-digested pNAN-OGG, yielding pBcmads1-GFP. For transforming into the *Bcmads1* mutant, the fragment was amplified from the constructed vector pBcmads1-GFP using the primer pair A5-F/A3-R. The *Bcmads1-GFP* fusion construct was then integrated into the *bcniiA* gene. Prior to microscopy, a conidial suspension was inoculated into PDB media and cultured for 0 h, 12 h and 24 h. The conidia, germinating conidia, and hypha were subsequently observed with a fluorescence microscope (Leica 2500, Germany).

### Southern blot analysis

The genomic DNA used for the Southern blot analysis was extracted from *B. cinerea* as described by Möller *et al*.^25^. The DNA probe was amplified and labeled by digoxin using digoxigenin-dUTP (Roche Applied Science). Hybridization was carried out at 65 °C in a buffer containing 6 × SSPE (1 × SSPE: 0.18 M NaCl, 10 mM NaPO_4_, and 2 mM EDTA [pH 7.7]), 5 × Denhardt’s solution (1 × Denhardt’s solution: 0.02% Ficoll 400, 0.02% polyvinylpyrrolidone, 0.02% bovine serum albumin), 0.5% SDS, and 100 μg of denatured salmon sperm DNA per milliliter. The DNA band with the hybridized probe was visualized using an enzyme immunoassay and enzyme catalyzed color reaction with NBT/BCIP (Roche Applied Science).

### Detection of gene expression

To detect the expression of the light-responsive genes, total RNA of 48-hour-old mycelium, which were respectively cultured under light and dark conditions, were extracted using Trizol reagent (Tiangen, Beijing). Samples containing 1 μg of RNA were treated with 1 U DNase I (Takara, Tokyo) for 30 min, and first-stand cDNA was synthesized using M-MLV reverse transcriptase (Promega, Madison, WI, U.S.A.). Quantitative real-time PCR was carried out using Step One plus real-time PCR system (AB Applied Biosystems, Foster City, CA, U.S.A.) using SYBR green PCR master mix. The relative expression levels were calculated using the 2^(−ΔCt)^ analysis method. Samples were normalized using actin (Bcin16g02020) and tubulin (Bcin01g08040) genes of *B. cinerea*.

### Two-dimensional (2D) gel electrophoresis of total protein

Total protein was extracted from 2-day-old mycelium that was cultured in PDB according to the method described by Li *et al*.^26^. For 2D gel electrophoresis, 500 μg of protein was loaded on an Immobiline DryStrip (pH 4 to 7, 13 cm; GE Healthcare, Piscataway, NJ, U.S.A.). The 2D electrophoresis and gel staining were carried out as previously described^27^. Images of stained gel were captured using a flatbed scanner (Amersham Biosciences, Uppsala, Sweden). Comparison of images between samples was performed using Image Master 2D Elite software (Amersham Biosciences). To account for experimental variation, three biological replicate gels resulting from three independent experiments were analyzed for the wild-type and mutant strains.

### In-gel digestion, mass spectrometry, and protein identification

In-gel digestion was performed as previously described^24^. Protein spots were excised from the 2D gels and destained with 50 mM NH_4_HCO_3_ in 50% (vol/vol) methanol for 1 h at 40 °C until the blue color of CBB R-250 was removed. After completely drying in a vacuum centrifuge, the excised, destained gel pieces were digested with 10 ng of trypsin per microliter at 37 °C for 16 h. Digested peptides were precipitated with 0.1% trifluoroacetic acid (TFA) in 50% acetonitrile, lyophilized and subsequently used for MS analysis using a MALDI-TOF/TOF mass spectrometer.

The precipitated peptides were resuspended in 1 mg mL^−1^ of α-cyano-4-hydroxycinnamic acid solution in 70% acetonitrile containing 0.1% TFA prior to being spotted onto the MALDI target plates. MS spectra were gathered with 400 laser shots per spectrum and MS/MS spectra were acquired with 1,500 laser shots per fragmentation spectrum. The 15 strongest peaks from each MS spectra were selected as precursor ions for the acquirement of the MS/MS fragmentation spectra. The 4000 spectra analyses were used for the generation of peak list files. The parameters were set to a signal-to-noise threshold of 10 and a minimum area of 100.

The MS data were searched against NCBInr protein database (version 20120107; 16,831,682 sequences and 5,781,564,572 residues) using Mascot. Search parameters were set as follows: fungi; proteolytic enzyme, trypsin; max missed cleavages, 1; fix modifications, carbamidomethyl (C); variable modifications, oxidation (M); peptide mass tolerance, 100 ppm; and fragment mass tolerance, 0.5 Da. Only significant hits as defined by Mascot probability analysis were considered.

### Virulence assays

The virulence of *B. cinerea* strains was assayed on apple fruit and tomato leaves. Conidia of wild-type B05.10 and mutants were collected from 2-week-old cultures grown on PDA plates and suspended in PDB at a final concentration of 5 × 10^4^ conidia per milliliter. Pre-wounded apple fruits were inoculated with a 10 μl droplet of a conidial suspension and incubated at 25 °C in enclosed plastic trays in order to maintain a high relative humidity (95%). Detached leaves from 4-week-old tomato plants were inoculated with 5 μl of a conidial suspension. The leaves were subsequently incubated in petri dishes at 25 °C. Disease symptoms were scored every day.

### Preparation of extracellular proteins

A suspension of 5 × 10^6^ conidia was inoculated into 100 ml Czapeck liquid medium. After culturing for 7 d, the filtrated supernatant was centrifuged at 20,000 × g at 4 °C for 1 h to remove impurities. The extracellular proteins were then extracted using the method previously described for total protein extraction^24^.

## Results

### *Bcmads1* encodes a MEF-type MADS-box transcription factor

**Figure 1:**
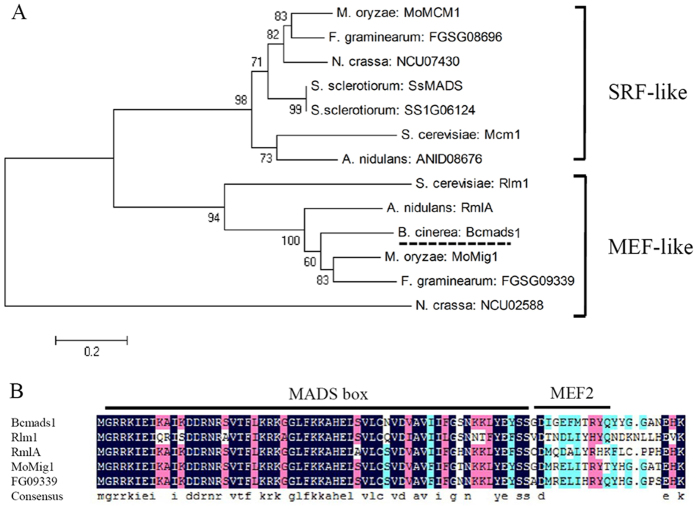
Phylogenetic analysis of Bcmads1 and homology of Bcmads1 to other MADS-box transcription factors. (**A**) Phylogenetic analysis of MADS-box transcription factors from a variety of fungi. (**B**) Alignment of the first 80 amino acids of different MADS-box transcription factors.

The Ensembl Fungi (http://fungi.ensembl.org/Botrytis_cinerea/Info/Index) database hosts the annotated genome sequence for *B. cinerea* strain B05.10. Within that genomic sequence, three genes are annotated as MADS-box transcription factor gene (Bcin05g04030, Bcin11g03800 and Bcin09g06140). The gene Bcin05g04030 encodes a MADS-box family transcription factor gene referred to as *Bcmads1* in the present study. The coding region of Bcmads1 is 2071 bp in length including 2 introns, and encoding a protein of 652 amino acids. Phylogenetic analysis indicates that the Bcmads1 protein clusters with the MEF-type class of MADS-box transcription factors, along with Rlm1 (*S. cerevisiae*), RmlA (*Aspergillus nidulans*), MoMig1 (*M. oryzae*), FG09339 (*Fusarium graminearum*), and NCU02558 (*Neurospora crassa*) (Fig. 1A). The amino acid sequence of Bcmads1 exhibits a considerable homology to the sequence of RmlA, MoMig1 and FG09339. The overall amino acid identity of Bcmads1 to RmlA, MoMig1, and FG09339 is 45.84%, 47.04%, and 45.04%, respectively. The specific motifs, including the 58-amino-acid MADS-box region and 11-amino-acid MEF2 region which determine the characteristics of transcription factors, were located near the N terminus. Results of alignment indicated that this region was more conserved than the other portions of the protein sequence. The first 80 amino acids of the Bcmads1 protein has 76.25% identity to RmlA, 87.5% identity to MoMig1, and 85% identity to FG09339 (Fig. 1B).

### Bcmads1 localizes to the nucleus

**Figure 2:**
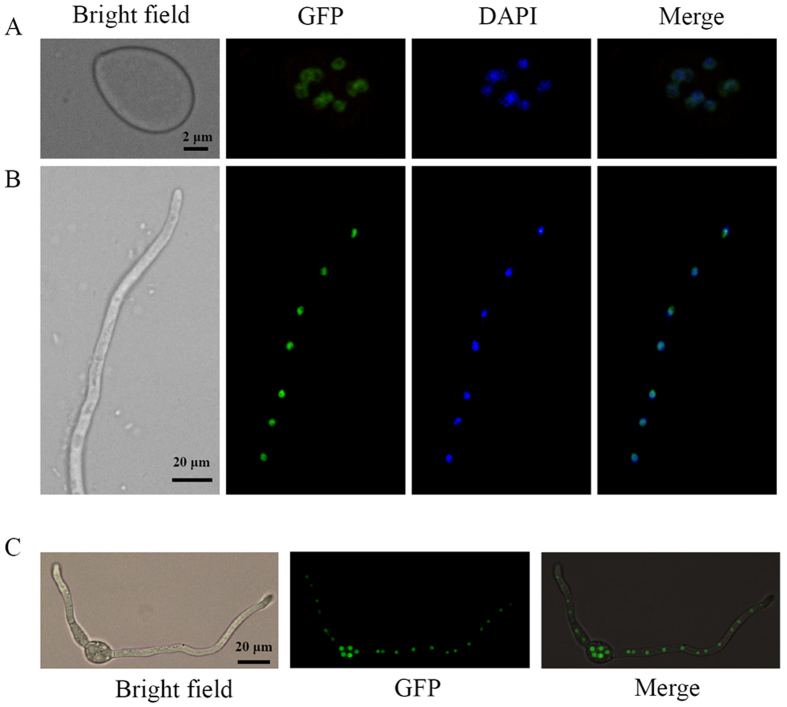
Subcellular localization of Bcmads1-eGFP. (**A**) Conidia obtained from the *Bcmads1-eGFP* transformant were stained with DAPI and observed using epifluorescence microscopy. (**B**) Hyphae (24 h) of the *Bcmads1-eGFP* transformant were stained with DAPI and observed using epifluorescence microscopy. (**C**) Germinating conidia (12 h) were observed using epifluorescence microscopy.

Nuclear localization is a defining characteristic of transcription factors. The localization of Bcmads1 was examined at three developmental stages: conidia, germinating conidia and young hyphae. The green fluorescence signal of the GFP-tagged Bcmads1 protein, as observed with fluorescence microscopy, was exclusively present in the nuclei of conidia (Fig. 2A), germinating conidia (Fig. 2C), and young hyphae (Fig. 2B). These data indicate that *Bcmads1* is constitutively expressed and localizes to the nucleus in *B. cinerea*. The observations made with fluorescence microscopy also indicate that the conidia and hyphae are both multinucleate.

### Generation of *Bcmads1* deletion mutants

To investigate the function of *Bcmads1*, knockout mutants of *Bcmads1* were generated by homologous recombination using a previously described method^24^ (Supplementary Fig. S1A). Three independent knockout mutants were identified by flank-spanning PCR and one round of single-spore isolation was subsequently conducted in order to exclude heterokaryons (Supplementary Fig. S1B). Result of Southern blot assay indicated that single homologous integration, without additional ectopic integration, occurred in the mutant of* Bcmads1* gene (*∆Bcmads1*) (Supplementary Fig. S1C). The three independent knockout mutants behaved identically as the mutant that was shown in this paper.

### *Bcmads1* is required for vegetative growth and sclerotia formation

**Figure 3:**
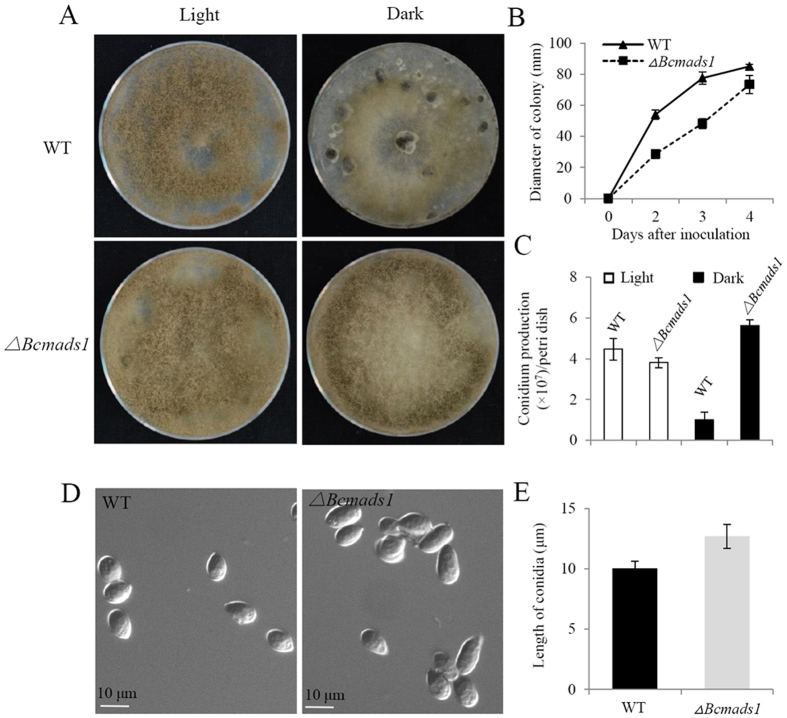
Conidiation and growth rate assays. (**A**) Examples of conidiation in the wild-type and *∆Bcmads1* strains cultured under light illumination or in darkness. Photographs were taken two weeks after inoculation on PDA plates. (**B**) Radial growth rate on PDA plates. Data represent the mean ± S.D. of three biological replicates. (**C**) Conidial production in wild-type and *∆Bcmads1* strains grown under light illumination or in darkness for seven days. (**D**) The morphology of wild-type and *∆Bcmads1* conidia. (**E**) Quantitation of conidial length.

Deletion of *Bcmads1* significantly reduced the vegetative growth of the mutant strains. The colony diameter of the mutant exhibited a 47% reduction at 2 dpi, relative to the wild-type strain and a 38% reduction at 3 dpi (Fig. 3B). The conidia that formed in the deletion mutant were more flat and larger than that of wild type (Fig. 3D,E). In the wild type B05.10, incubation in light induced the formation of conidia while prolonged incubation in darkness resulted in the formation of sclerotia. In the *∆Bcmads1* knockout mutant, however, the light-induced formation of conidia was similar to the wild-type strain but the mutant lost the ability of producing sclerotia upon incubation in darkness (Fig. 3A). The production of conidia in the *∆Bcmads1* mutant was even higher in the dark than it was in the light (Fig. 3C). After reintroducing the *Bcmads1* gene back into the knockout mutant, the ability to produce sclerotia in the complemented mutant strain was restored (Supplementary Fig. S4A). These results implied that *Bcmads1* may play an important role in response to light.

### The lesion expansion rate of *Bcmads1* mutant is reduced

**Figure 4:**
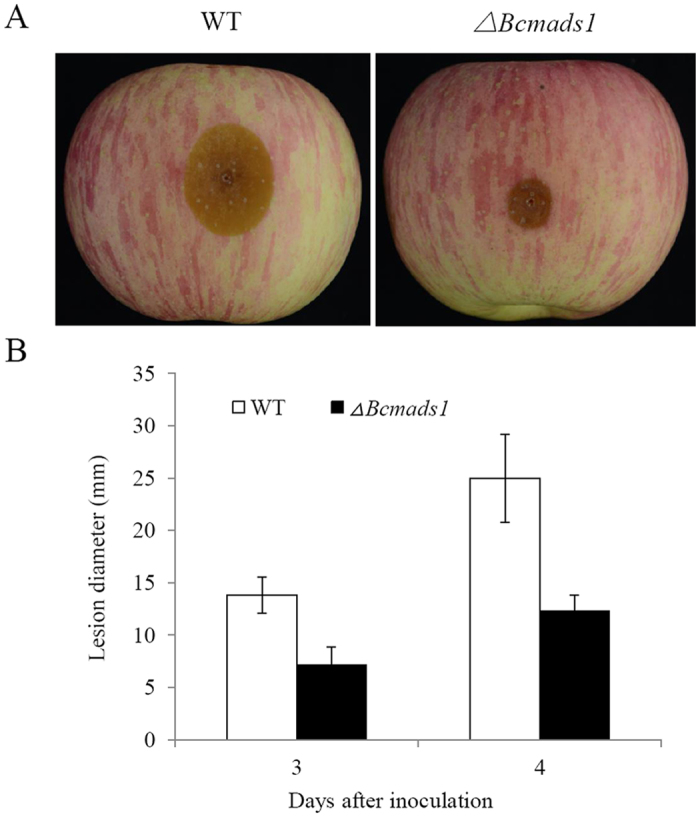
Virulence of wild-type and *∆Bcmads1* strains of *B. cinerea.* (**A**) The lesions generated by wild-type and *∆Bcmads1* strains in apples at 4 days after inoculation. (**B**) Lesion diameter on apple fruits at 3 and 4 days post inoculation (dpi). Data presented represent the mean ± S. D. (n = 10).

Pathogenicity assays were carried out on apple fruit to determine how the deletion of *Bcmads1* affected the virulence of *∆Bcmads1*. The lesions produced by the *∆Bcmads1* mutant were significantly smaller (approximately 50%) than lesions produced by the wild-type strain. Four days after inoculation, the lesion size resulting from the inoculation of fruit with *∆Bcmads1* was approximately 12 mm compared to 25 mm in fruit inoculated with the wild type strain (Fig. 4). The lesions produced on apple fruit by the complemented mutant strain were similar to those produced by the wild-type strain (Fig. S4B).

### *Bcmads1* regulates the expression of light-responsive genes

**Figure 5:**
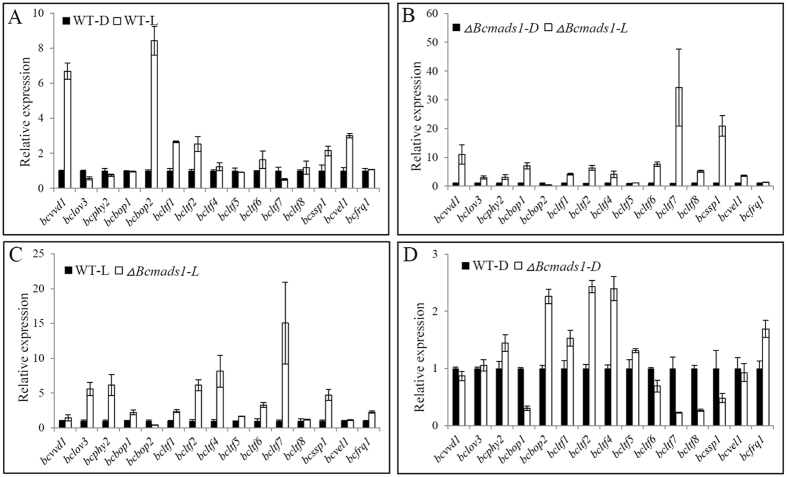
Relative expression of light-responsive genes in wild-type and *∆Bcmads1* strains under light or dark conditions. (**A**) Relative expression of light-responsive genes in the wild-type strain under light and dark conditions. (**B**) Relative expression of light-responsive genes in the *∆Bcmads1* strain under light and dark conditions. (**C**) Relative expression of light-responsive genes in wild-type and *∆Bcmads1* strains under light conditions. (**D**) Relative expressions of light-responsive genes in wild-type and *∆Bcmads1* strains under dark conditions. -D, dark condition; -L, light condition. Relative expression data was obtained by RT-qPCR analysis. Data presented represent the mean ± S.D. (n = 3).

The knockout mutant *∆Bcmads1* was unable to form sclerotia, which were exclusively induced in the absence of light in the wild-type strain. In order to further characterize the impact of *Bcmads1* on the light response of *B. cinerea*, the expression of multiple light-responsive genes was examined. Specifically, we compared the expression in the wild-type strain and the *∆Bcmads1* mutant of the photoreceptor-encoding genes *bcvvd1, bclov3, bcphy2, bcbop1* and *bcbop2*^28^,^29^,^30^; the light-induced genes encoding transcription factors *bcltf1, bcltf2, bcltf4, bcltf5, bcltf6, bcltf7* and *bcltf8*^30^; the *bcssp1* gene encoding a sclerotium-specific marker protein^31^,^32^; the VELVET superfamily gene *bcvel1*^33^; and finally, the circadian clock gene *bcfrq1*^29^,^34^. The wild-type and *∆Bcmads1* were cultured under continuous white light illumination or darkness for 48 h. Subsequently, RNA was extracted from the two strains and gene expression was analyzed. In the B05.10 wild-type strain, the expression of the photoreceptor genes *bcvvd1* and *bcbop2*, the light-responsive transcription factor genes *bcltf1, bcltf2*, and *bcltf6*, the sclerotia specific gene *bcssp1,* and the VELVET superfamily gene *bcvel1* were significantly higher under light condition comparing to that in darkness (Fig. 5A). In the *∆Bcmads1* mutant strain, the expression of *bclov3, bcphy2, bcbop1*, and *bcltf7* was higher under light illumination, while the expression level of *bcbop2* was lower under light condition, relative to expression levels in darkness (Fig. 5B). Overall, the expression of photoreceptor genes (except *bcbop2*) and transcription factor genes (except *bcltf8*) in the *∆Bcmads1* mutant was much higher than compared to the wild-type strain under light conditions. This was especially evident for *bclov3, bcphy2, bcltf2* and *bcltf4* where their expression levels were more than 5 times higher than in the wild-type strain, and the expression of *bcltf7* was more than 10 times higher than in wild type strain (Fig. 5C). Under dark conditions, the expression of *bcssp1*, which was a marker gene of sclerotium development^31^,^32^, was decreased in the *∆Bcmads1* mutant (Fig. 5D). Interestingly, although *Bcmads1* presumably played an important role in regulating the expression of light responsive genes, the expression of *Bcmads1* was only slightly affected by light illumination (Supplementary Fig. S2).

### Proteomic identification of the potential downstream targets of Bcmads1

**Figure 6:**
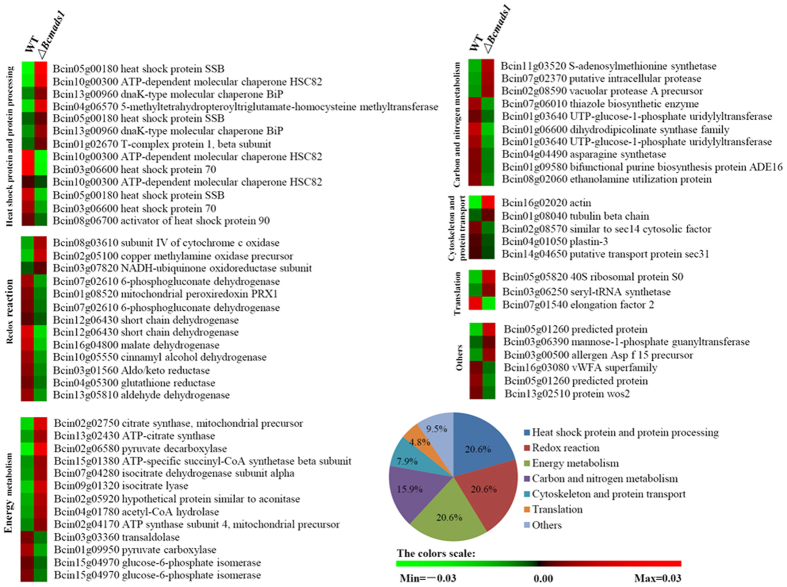
Quantitative analysis of protein abundance in the potential targets of *Bcmads1*. The differential abundance of proteins in the wild-type and *∆Bcmads1* strains determined by two-dimensional (2D) gel electrophoresis. The proteins were classified based on their putative functions. A bright red color indicates a high percentage of the protein spot volume and a bright green color represents a low percentage of the protein spot volume.

A comparative analysis of the proteome of the wild-type and *∆Bcmads1* mutant strain was conducted using two-dimensional (2D) gel electrophoresis. Total proteins were extracted from the mycelia of 48-hour old cultures grown in PDB. The analysis was performed using three biological replicates and the gel images were analyzed using Image Master 2D Elite software. Approximately 1,000 reproducible protein spots were detected on CBB-stained gels after discarding faint spots and spots with undefined shapes and areas (Supplementary Fig. S3). Spots showing statistically significant (P < 0.05) changes in relative abundance (greater than 2-fold) were designated as differentially abundant. Based on this standard, a total of 67 differentially abundant proteins were detected. These proteins were excised from the gel and submitted to Q-TOF MS/MS analysis, resulting in the identification of 63 spots with MOWSE scores significantly higher than the threshold (P < 0.05). Out of these 63 proteins, 29 proteins had higher levels of abundance in the *∆Bcmads1* mutant and 34 had lower levels of abundance relative to the wild-type strain. The differentially abundant proteins were placed into 7 categories based on their function: heat shock protein and protein processing (13 proteins, 20.6%); redox reaction (13 proteins, 20.6%); tricarboxylic acid cycle and energy metabolism (13 proteins, 20.6%); carbon and nitrogen metabolism (10 proteins, 15.9%); cytoskeleton and protein transport (5 proteins, 7.9%); translation (3 proteins, 4.8%); and others (6 proteins, 9.5%) (Fig. 6).

### *Bcsec14* and *Bcsec31* are required for pathogenicity and protein secretion in *B. cinerea*

**Figure 7:**
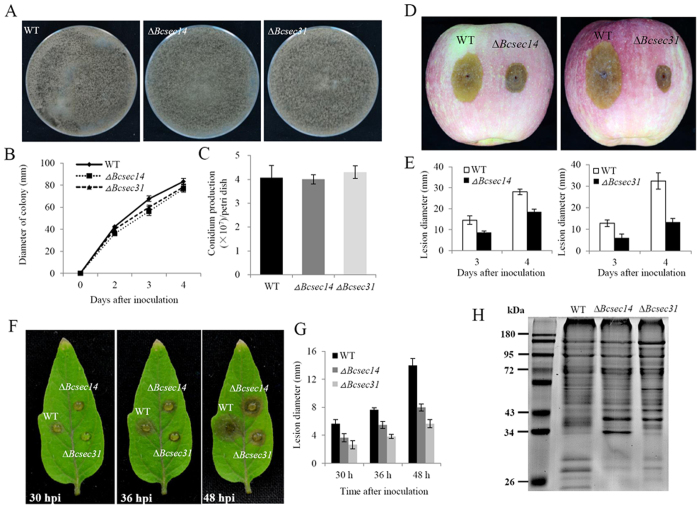
Determination of the function of *Bcsec14* and *Bcsec31*. (**A**) The colony phenotype of wild-type, *∆Bcsec14,* and *∆Bcsec31* strains two weeks after inoculation on PDA plates. (**B**) Radial growth rate on PDA plates. Data represent the mean ± S.D. of three biological replicates. (**C**) Quantitation of conidial production. Data represent the mean ± S.D. of three biological replicates. (**D**) Virulence of wild-type, *∆Bcsec14,* and *∆Bcsec31* strains on apple fruits. Images are of lesions 4 days post inoculation (dpi). (**E**) Diameters of lesions induced by the wild-type, *∆Bcsec14,* and *∆Bcsec31*strains at 3 and dpi. Data presented represent the mean ± S.D. (n = 10). (**F**) Virulence of wild-type, *∆Bcsec14,* and *∆Bcsec31* strains on detached tomato leaves. (**G**) Quantitation of lesion diameter of tomato leaves. Data presented represent the mean ± S.D. of three biological replicates. (**H**) SDS-PAGE of the secreted proteins obtained from the wild-type, *∆Bcsec14,* and *∆Bcsec31* strains. Five μg of proteins from the samples were loaded into each lane. Molecular weight markers are visualized in the far left lane.

Among the differentially abundant proteins, spot 57 and spot 67 were homologs of yeast sec14 and sec31^35^,^36^, respectively, which have been demonstrated to play a role in protein secretion. In our study, these two proteins were named *Bcsec14* and *Bcsec31*, respectively. The abundance of Bcsec14 and Bcsec31 were significantly reduced in the *∆Bcmads1* mutant (Fig. 6). Our previous study indicated that protein secretion plays an important role in the development and pathogenicity of *B. cinrea*^24^. Therefore, the function of these two genes was further examined. Deletion mutants of *Bcsec14* and *Bcsec31* were generated using homologous recombination (Supplementary Fig. S5). The deletion of these two genes, separately, slightly reduced the growth rate (Fig. 7B), but did not affect colony morphology (Fig. 7A), conidia production (Fig. 7C) and the conidial germination (Fig. S6A,B). In yeast and other model species, the homologues of *Bcsec14* are required for transport of secretory proteins from the Golgi complex^37^,^38^. Homologues of *Bcsec31* are components of the outer layer of the COPII coat of vesicles that are required for the trafficking of proteins from the endoplasmic reticulum to the Golgi complex^36^. In order to explore the function of *Bcsec14* and *Bcsec31* in protein secretion, the extracellular proteins of the mutants were examined by SDS-PAGE and compared with extracellular protein profiles obtained from the wild-type strain. Results indicated that deletion of these two genes, separately, altered the composition of extracellular proteins and the number of low molecular weight extracellular proteins decreased in the mutant strains (Fig. 7H). The virulence of *∆Bcsec14* and *∆Bcsec31* were assayed on apple fruits and detached tomato leaves. The overall lesion size and expansion rates were significantly reduced in the two mutants, relative to the wild-type strain (Fig. 7D). At four days post-inoculation (dpi), the lesion diameter generated by *∆Bcsec14* and *∆Bcsec31* was reduced by 34% and 58%, respectively, relative to the wild-type strain (Fig. 7E). The result obtained on detached tomato leaves was consistent with results on apples. The deletion of *Bcsec14* and *Bcsec31* significantly delayed the occurrence of symptoms. The wild-type strain produced an obvious lesion at 24 hour post-inoculation (hpi), however, the mutants *∆Bcsec14* and *∆Bcsec31* only produced an obvious lesion at 30 hpi and 36 hpi, respectively (Fig. 7E,F). These results demonstrate that both *Bcsec14* and *Bcsec31* are required for the pathogenicity of *B. cinerea* and that *Bcsec31* had a greater influence on the virulence of *B. cinerea* than *Bcsec14.*

## Discussion

The MADS-box family of transcription factors is widely present and highly conserved in eukaryotes and has been demonstrated to be crucial for numerous life processes in a variety of model organisms. In the present study, the regulatory network of the MADS-box transcription factor *Bcmads1* in *B. cinerea* was characterized and it was demonstrated that *Bcmads1* plays an important role in vegetative growth, sclerotia production, and pathogenesis.

Two major classes of MADS-box family transcription factors are the SRF-type and MEF-type. A phylogenetic analysis using a variety of MADS-Box protein sequences indicated that Bcmads1 clustered with the MEF-type, along with Rlm1 (*S. cerevisiae*), RmlA (*A. nidulans*), MoMig1 (*M. oryzae*) and FG09339 (*F. graminearum*). In *M. oryzae*, the growth rate of a *MoMig1* mutant was similar to the wild-type, but the mutant exhibited a reduction in aerial hyphal growth and conidiation^18^. In contrast, in the current study, the deletion of *Bcmads1* in *B. cinerea* resulted in a different phenotype than the deletion of the *MoMig1* gene in *M. oryzae*. Vegetative growth in the *∆Bcmads1* mutant was reduced, relative to the wild-type B05.10 strain, but the mutant formed more aerial hyphal growth when grown on PDA plates and conidia in the mutant were bigger than in the wild-type strain. The function of MADS-box transcription factors in the virulence of plant pathogens varies among different fungi. In *M. oryzae, MoMig1* was important for overcoming plant defense response and for the differentiation of secondary infectious hyphae in host cells^18^, whereas it was not required for virulence in *U. maydis*^20^. Virulence assays conducted in our study suggest that *Bcmads1* is important for the virulence of *B. cinerea*. The lesion diameter of the *∆Bcmads1* mutant was reduced by approximately 50% as compared to the wild-type strain. Negative effects of deletion of *Bcmads1* on vegetative growth might largely contribute to the reduction of lesion sizes in apple fruits.

*B. cinerea* reproduces asexually by forming conidia which serve as the main source of primary inoculum. The fungus also produces dark pigmented sclerotia which serve as survival structures during adverse environmental conditions such as over-wintering or they serve as the female parent during sexual reproduction. The formation of conidia vs. sclerotia in *B. cinerea* occurs in a light-dependent pattern: white light induces the formation of conidia while darkness induces the formation of sclerotia^39^. Interestingly, the deletion of *Bcmads1* changed the response of *B. cinerea* to light illumination. In contrast to the wild-type strain, the *∆Bcmads1* mutant was defective in producing sclerotia under dark condition. Light is a vital environmental cue that is capable of modulating the physiology of an organism^29^. Recently, light has been recognized as an important modulator of many fundamental processes in filamentous fungi, such as development, metabolism, and pathogenesis^40^,^41^,^42^,^43^. A previous study reported that a ‘white collar’ complex in *B. cinerea*, comprised of Bcwcl1 and Bcwcl2 transcription factors, mediated white light-induced gene expression, functioned as a repressor of conidiation, is required for tolerating excessive illumination, and is needed to achieve full virulence in the presence of light^29^. Schumacher *et al*. reported that the *bcltf1* GATA transcription factor in *B. cinerea* plays an important role in light-dependent differentiation, virulence, establishing the equilibrium of reactive oxygen species, and secondary metabolism^30^. Similar to Bcwcl1, Bcmads1 also acts as a suppressor of conidiation in the absence of light. Similar to the wild-type strain, light can also induce the formation of conidia in the *∆Bcmads1* mutant; however, under dark conditions the mutant is unable to form sclerotia. The *∆Bcmads1* mutant induces conidiation rather than the formation of sclerotia in the darkness. Sclerotia are very important for the survival of *B. cinrea* in the natural environment as it provides protection from a wide array of adverse environmental conditions. Sclerotia can germinate either vegetatively to produce mycelia and conidia, or initiate the sexual cycle. By examining gene expression, we found that the deletion of *Bcmads1* perturbed the expression pattern of several important light-responsive genes. This implies that *Bcmads1* is involved in the light signaling pathway in *B. cinerea*. Deletion of *Bcmads1* enhanced the expression of several light-responsive genes under light illumination. The expression of genes encoding photoreceptors (*bcvvd1, bclov3, bcphy2* and *bcbop1*), light-responsive transcription factors (*bcltf1, bcltf2, bcltf4, bcltf5, bcltf6* and *bcltf7*), a sclerotia-specific protein (*bcssp1*), and a circadian rhythm component FRQ (*bcfrq1*) were all significantly upregulated under white light illumination in the *∆Bcmads1* mutant. Among the photoreceptors, bcvvd1 is a potential blue light receptor, bcphy2 is red light-sensing phytochrome and bcbop1 is an opsin which is putative green light receptor^28^,^29^,^30^. We hypothesize that deletion of *Bcmads1* results in that *B. cinerea* has to generate more photoreceptors to percept enough light illumination. The increased expression of photoreceptor genes may be driven by the transcription factor that also had increased expression in the *∆Bcmads1* mutant. In darkness, the expression of *bcssp1* was significantly decreased in the *∆Bcmads1* mutant and it indicated that the formation of sclerotia in the absence of light was mediated by *Bcmads1*.

In order to identify the potential targets of *Bcmads1*, proteome profiles in wild-type and the *∆Bcmads1* mutant were analyzed. Using tandem mass spectrometry (MS/MS) analysis, 63 proteins that were differentially abundant in the *∆Bcmads1* mutant, relative to the wild-type strain, were successfully identified. The light responsive proteins, however, were not detected which could perhaps be attributed to the limitations in the sensitivity of this method. Among the differentially abundant proteins, homologues of the cytosolic factor Sec14 and the transport protein Sec31 were closely associated with protein secretion in *B. cinerea* (Fig. 7H). During the process of invading host tissues, *B. cinerea* secretes an arsenal of virulence factors into the extracellular environment to facilitate the infection process^7^,^8^,^9^,^10^. Our previous study also demonstrated that protein secretion plays an important role in the pathogenesis of *B. cinerea*^24^. Sec14 protein catalyzes the transfer of phosphatidylinositol and phosphatidylcholine between membranes and is required for the transport of secretory proteins from the Golgi complex^44^. In *S. cerevisiae*, Sec14 is essential for protein secretion and viability^45^,^46^. In the human pathogen *Cryptococcus neoformans*, the Sec14 protein is specifically required for secretion of phospholipase B1, which is essential for the dissemination of *C. neoformans* to the central nervous system^47^. Sec31 is a component of the outer layer of the COPII coat of vesicles and is involved in ER-to-Golgi trafficking^48^. In the present study, the virulence of *Bcsec14* and *Bcsec31* deletion mutants was significantly reduced and the secretion of extracellular proteins in these mutants decreased relative to the wild-type strain. These results indicate that *Bcsec14* and *Bcsec31* play a crucial role in protein secretion and pathogenicity. Furthermore, it implies that protein secretion is an important process through which *Bcmads1* regulates differentiation and pathogenicity in *B. cinerea*. No significant difference was observed, however, in the expression pattern of these two genes in the wild-type strain and the *∆Bcmads1* mutant; implying that *Bcmads1* may influence protein secretion indirectly.

In conclusion, all these data indicate that *Bcmads1* is an important modulator in pathogenesis and light response. *Bcmads1* can regulate the expression pattern of some important light-responsive genes which maybe in turn exert impact on the photomorphogenesis of *B. cinerea. Bcsec14* and *Bcsec31*, whose protein abundance can be significantly modulated by *Bcmads1*, contribute to the protein secretion and pathogenicity of *B. cinerea*.

## Additional Information

**How to cite this article**: Zhang, Z. *et al*. The MADS-Box transcription factor Bcmads1 is required for growth, sclerotia production and pathogenicity of *Botrytis cinerea. Sci. Rep.* **6**, 33901; doi: 10.1038/srep33901 (2016).

## Supplementary Material

Supplementary Information

## References

[b1] Williamson B., Tudzynski B., Tudzynski P. & van Kan J. A. L. *Botrytis cinerea*: The cause of grey mould disease. Mol. Plant Pathol. 8, 561–580 (2007). 20507522 10.1111/j.1364-3703.2007.00417.x

[b2] Dean P. . The top 10 fungal pathogens in molecular plant pathology. Mol. Plant Pathol. 13, 414–430 (2012). 22471698 10.1111/j.1364-3703.2011.00783.x PMC6638784

[b3] Xu J. R. MAP kinases in fungal pathogens. Fungal Genet. Biol. 31, 137–152 (2000). 11273677 10.1006/fgbi.2000.1237

[b4] Segmüller N., Ellendorf U., Tudzynski B. & Tudzynski P. BcSAK1, a stress-activated MAP kinase is involved in vegetative differentiation and pathogenicity in *Botrytis cinerea*. Eukaryot. Cell 6, 211–221 (2007). 17189492 10.1128/EC.00153-06 PMC1797955

[b5] Segmüller N. . NADPH Oxidases are involved in differentiation and pathogenicity in *Botrytis cinerea*. Mol. Plant-Microbe Interact. 21, 808–819 (2008). 18624644 10.1094/MPMI-21-6-0808

[b6] Siegmund U., Marschall R. & Tudzynski P. BcNoxD, a putative ER protein, is a new component of the NADPH oxidase complex in *Botrytis cinerea*. Mol. Microbiol. 95, 988–1005 (2015). 25402961 10.1111/mmi.12869

[b7] ten Have A., Mulder W., Visser J. & van Kan J. A. L. The endopoly-galacturonase gene Bcpg1 is required for full virulence of *Botrytis cinerea*. Mol. Plant-Microbe Interact. 11, 1009–1016 (1998). 9768518 10.1094/MPMI.1998.11.10.1009

[b8] Valette-Collet O., Cimerman A., Reignault P., Levis C. & Boccara M. Disruption of *Botrytis cinerea* pectin methylesterase gene *Bcpme1* reduces virulence on several host plants. Mol. Plant-Microbe Interact. 16, 360–367 (2003). 12744465 10.1094/MPMI.2003.16.4.360

[b9] Schouten A., van Baarlen P. & van Kan J. A. L. Phytotoxic Nep1-like proteins from the necrotrophic fungus *Botrytis cinerea* associate with membranes and the nucleus of plant cells. New Phytol. 177, 493–505 (2008). 18028294 10.1111/j.1469-8137.2007.02274.x

[b10] Frías M., González C. & Brito N. BcSpl1, a cerato-platanin family protein, contributes to *Botrytis cinerea* virulence and elicits the hypersensitive response in the host. New Phytol. 192, 483–495 (2011). 21707620 10.1111/j.1469-8137.2011.03802.x

[b11] Shore P. & Sharrocks A. D. The MADS-box family of transcription factors. Eur. J. Biochem. 229, 1–13 (1995). 7744019 10.1111/j.1432-1033.1995.tb20430.x

[b12] Messenguy F. & Dubois E. Role of MADS box proteins and their cofactors in combinatorial control of gene expression and cell development. Gene 316, 1–21 (2003). 14563547 10.1016/s0378-1119(03)00747-9

[b13] Watanabe Y., Irie K. & Matsumoto K. Yeast RLM1 encodes a serum response factor-like protein that may function downstream of the Mpk1 (Slt2) mitogen-activated protein kinase pathway. Mol. Cell. Biol. 15, 5740–5749 (1995). 7565726 10.1128/mcb.15.10.5740 PMC230825

[b14] Dodou E. & Treisman R. The *Saccharomyces cerevisiae* MADS-Box transcription factor Rlm1 as a target for the Mpk1 mitogen-activated protein kinase pathway. Mol. Cell. Biol. 17, 1848–1859 (1997). 9121433 10.1128/mcb.17.4.1848 PMC232032

[b15] Nadal Ed E., Casadome L. & Posas F. Targeting the MEF2-like transcription factor Smp1 by the stress-activated Hog1 mitogen-activated protein kinase. Mol. Cell. Biol. 23, 229–237 (2003). 12482976 10.1128/MCB.23.1.229-237.2003 PMC140668

[b16] Messenguy F. & Dubois E. Genetic evidence for a role for MCM1 in the regulation of arginine metabolism in *Saccharomyces cerevisiae*. Mol. Cell. Biol. 13, 2586–2592. (1993). 8455631 10.1128/mcb.13.4.2586-2592.1993 PMC359592

[b17] Messenguy F. & Dubois E. Regulation of arginine metabolism in *Saccharomyces cerevisiae*: a network of specific and pleiotropic proteins in response to multiple environmental signals. Food Technol. Biotechnol. 38, 277–285 (2000).

[b18] Mehrabi R., Ding S. & Xu J. R. MADS-box transcription factor Mig1 is required for infectious growth in *Magnaporthe grisea*. Eukaryot. Cell 7, 791–799 (2008). 18344407 10.1128/EC.00009-08 PMC2394974

[b19] Zhou X. Y. . A MADS-box transcription factor MoMcm1 is required for male fertility, microconidium production and virulence in *Magnaporthe oryzae*. Mol. Microbiol. 80, 33–53 (2011). 21276092 10.1111/j.1365-2958.2011.07556.x

[b20] Krueger J., Aichinger C., Kahmann R. & Boelker M. A MADS-box homologue in *Ustilago maydis* regulates the expression of pheromone-inducible genes but is nonessential. Genetics 147, 1643–1652 (1997). 9409827 10.1093/genetics/147.4.1643 PMC1208337

[b21] Yang C. . The MADS-box transcription factor FgMcm1 regulates cell identity and fungal development in *Fusarium graminearum*. Environ. Microbiol. 17, 2762–2776 (2015). 25627073 10.1111/1462-2920.12747

[b22] Qu X. Y. . MADS-Box transcription factor SsMADS is involved in regulating growth and virulence in *Sclerotinia sclerotiorum*. Int. J. Mol. Sci. 15, 8049–8062 (2014). 24815067 10.3390/ijms15058049 PMC4057718

[b23] Schumacher J. Tools for *Botrytis cinerea*: New expression vectors make the gray mold fungus more accessible to cell biology approaches. Fungal Genet. Biol. 49, 483–497 (2012). 22503771 10.1016/j.fgb.2012.03.005

[b24] Zhang Z. Q., Qin G. Z., Li B. Q. & Tian S. P. Knocking out Bcsas1 in *Botrytis cinerea* impacts growth, development, and secretion of extracellular proteins, which decreases virulence. Mol Plant-Microbe Interact. 27, 590–600 (2014). 24520899 10.1094/MPMI-10-13-0314-R

[b25] Möller E. M., Bahnweg G., Sandermann H. & Geiger H. H. A simple and efficient protocol for isolation of high molecular weight DNA from filamentous fungi, fruit bodies and infected plant tissues. Nucleic Acids Res. 20, 6115–6116 (1992). 1461751 10.1093/nar/20.22.6115 PMC334490

[b26] Li B. Q., Lai T. F., Qin G. Z. & Tian S. P. Ambient pH stress inhibits spore germination of *Penicillium expansum* by impairing protein synthesis and folding: a proteomic-based study. J. Proteome Res. 9, 298–307 (2010). 19951004 10.1021/pr900622j

[b27] Qin G. Z., Tian S. P., Chan Z. L. & Li B. Q. Crucial Role of Antioxidant Proteins and Hydrolytic Enzymes in Pathogenicity of *Penicillium expansum*. Mol. Cell. Proteomics 6, 425–438 (2007). 17194899 10.1074/mcp.M600179-MCP200

[b28] Idnurm A., Verma S. & Corrochano L. M. A glimpse into the basis of vision in the kingdom Mycota. Fungal Genet. Biol. 47, 881–892 (2010). 20451644 10.1016/j.fgb.2010.04.009 PMC2950209

[b29] Canessa P., Schumacher J., Hevia M. A., Tudzynski P. & Larrondo L. F. Assessing the effects of light on differentiation and virulence of the plant pathogen *Botrytis cinerea*: characterization of the White Collar complex. Plos One 8, e84233 (2013). 10.1371/journal.pone.0084223 PMC3877267 24391918

[b30] Schumacher J., Simon A., Cohrs K. C., Viaud M. & Tudzynski P. The transcription factor BcLTF1 regulates virulence and light responses in the necrotrophic plant pathogens *Botrytis cinerea*. PLoS Genet. 10, e1004040 (2014). 24415947 10.1371/journal.pgen.1004040 PMC3886904

[b31] Li M. & Rollins J. A. The development-specific protein (Ssp1) from *Sclerotinia sclerotiorum* is encoded by a novel gene expressed exclusively in sclerotium tissues. Mycologia, 101, 34–43 (2009). 19271669 10.3852/08-114

[b32] Li M. & Rollins J. A. The development-specific *ssp1* and *ssp2* genes of *Sclerotinia sclerotiorum* encode lectins with distinct yet compensatory regulation. Fungal Genet. Biol. 47, 531–538 (2010). 20350614 10.1016/j.fgb.2010.03.008

[b33] Schumacher J. . Natural variation in the VELVET gene *bcvel1* affects virulence and light-dependent differentiation in *Botrytis cinerea*. Plos One 7, e47840 (2012). 23118899 10.1371/journal.pone.0047840 PMC3485325

[b34] Baker C. L., Loros J. J. & Dunlap J. C. The circadian clock of *Neurospora crassa*. FEMS Microbiol. Rev. 36, 95–110 (2012). 21707668 10.1111/j.1574-6976.2011.00288.x PMC3203324

[b35] Monteoliva L., Sánchez M., Pla J., Gil C. & Nombela C. Cloning of *Candida albicans* SEC14 gene homologue coding for a putative essential function. Yeast 12, 1097–1105 (1996). 8896277 10.1002/(SICI)1097-0061(19960915)12:11%3C1097::AID-YEA990%3E3.0.CO;2-E

[b36] Koreishi M., Yu S., Oda M., Honjo Y. & Satoh A. CK2 phosphorylates Sec31 and regulates ER-to Golgi trafficking. Plos One 8, e54382 (2013). 23349870 10.1371/journal.pone.0054382 PMC3548793

[b37] Bankaitis V. A., Malehorn D. E., Erm S. D. & Greene R. The *Saccharomyces cerevisiae* SEC14 gene encodes a cytosolic factor that is required for transport of secretory proteins from the yeast Golgi complex. J. Cell Biol. 108, 1271–1281 (1989). 2466847 10.1083/jcb.108.4.1271 PMC2115512

[b38] Curwin A. J., Fairn G. D. & McMaster C. R. Phospholipid transfer protein Sec14 is required for trafficking from endosomes and regulates distinct trans-Golgi export pathways. J. Biol. Chem. 284, 7364–7375 (2009). 19129178 10.1074/jbc.M808732200 PMC2652273

[b39] Tan K. K. & Epton H. A. S. Effect of light on the growth and sporulation of *Botrytis cinerea*. Trans. Bri. Mycol. Soc. 61, 147–157 (1973).

[b40] Purschwitz J., Muller S., Kastner C. & Fischer R. Seeing the rainbow: light sensing in fungi. Curr. Opin. Microbiol. 9, 566–571 (2006). 17067849 10.1016/j.mib.2006.10.011

[b41] Herrera-Estrella A. & Horwitz B. A. Looking through the eyes of fungi: molecular genetics of photoreception. Mol. Microbiol. 64, 5–15 (2007). 17376067 10.1111/j.1365-2958.2007.05632.x

[b42] Idnurm A. & Crosson S. The photobiology of microbial pathogenesis. PLoS Pathog. 5, e1000470 (2009). 19956669 10.1371/journal.ppat.1000470 PMC2777379

[b43] Zhu P. . Exploitable regulatory effects of light on growth and development of Botrytis cinerea. J. Plant Pathol. 95, 509–517. (2013).

[b44] Monteoliva L., Sánchez M., Pla J., Gil C. & Nombela C. Cloning of *Candida albicans* SEC14 gene homologue coding for a putative essential function. Yeast 12, 1097–1105 (1996). 8896277 10.1002/(SICI)1097-0061(19960915)12:11%3C1097::AID-YEA990%3E3.0.CO;2-E

[b45] Bankaitis V. A., Malehorn D. E., Erm S. D. & Greene R. The *Saccharomyces cerevisiae* SEC14 gene encodes a cytosolic factor that is required for transport of secretory proteins from the yeast Golgi complex. J. Cell Biol. 108, 1271–1281 (1989). 2466847 10.1083/jcb.108.4.1271 PMC2115512

[b46] Curwin A. J., Fairn G. D. & McMaster C. R. Phospholipid transfer protein Sec14 is required for trafficking from endosomes and regulates distinct trans-Golgi export pathways. J. Biol. Chem. 284, 7364–7375 (2009). 19129178 10.1074/jbc.M808732200 PMC2652273

[b47] Chayakulkeeree M. . SEC14 is a specific requirement for secretion of phospholipase B1 and pathogenicity of *Cryptococcus neoformans*. Mol. Microbiol. 80, 1088–1101 (2011). 21453402 10.1111/j.1365-2958.2011.07632.x PMC3418542

[b48] Koreishi M., Yu S., Oda M., Honjo Y. & Satoh A. CK2 phosphorylates Sec31 and regulates ER-to Golgi trafficking. Plos One 8, e54382 (2013). 23349870 10.1371/journal.pone.0054382 PMC3548793

