# Supplementary material for: The MADS-Box transcription factor Bcmads1 is required for growth, sclerotia production and pathogenicity of Botrytis cinerea
Source: Sci Rep. 2016 Sep 23;6:33901. doi: 10.1038/srep33901 (PMC5034256; doi:10.1038/srep33901)
Supplement: Supplementary Information [file srep33901-s1.doc]

# Supplementary Information

# The MADS-Box transcription factor Bcmads is required for growth, sclerotia production and pathogenicity of *Botrytis cinerea*

Zhanquan Zhang, Hua Li, Guozheng Qin, Chang He, Boqiang Li, Shiping Tian

**Fig. S1. Diagrammatic representation of the construction and generation of the *Bcmads* mutant.** A. Replacement strategy for deletion of *Bcmads*. Flank L and Flank R were amplified from genomic DNA of *B. cinerea* using the primer pairs ML-F/ML-R and MR-F/MR-R, respectively. Flank L and Flank R were cloned into the upstream and downstream site of the hygromycin resistance cassette of pLOB7. B. Detection of the mutant by flank-spanning PCR using the primer pairs MJ-F/MJ-R. C. Southern blot analysis of the wild-type strain and the *Bcmads* deletion mutant. Genomic DNA was digested with *Hind*III and *Xho*I, separated in an agarose gel, blotted, and hybridized with a probe (Flank L labeled with digoxigenin). Primer binding sites are indicated by arrows and the probe is indicated by the dotted line. K, *Kpn*I; S, *Sac*I; N, *Nar*I; A, *Asc*I; H, *Hind*III; X, *Xho*I.


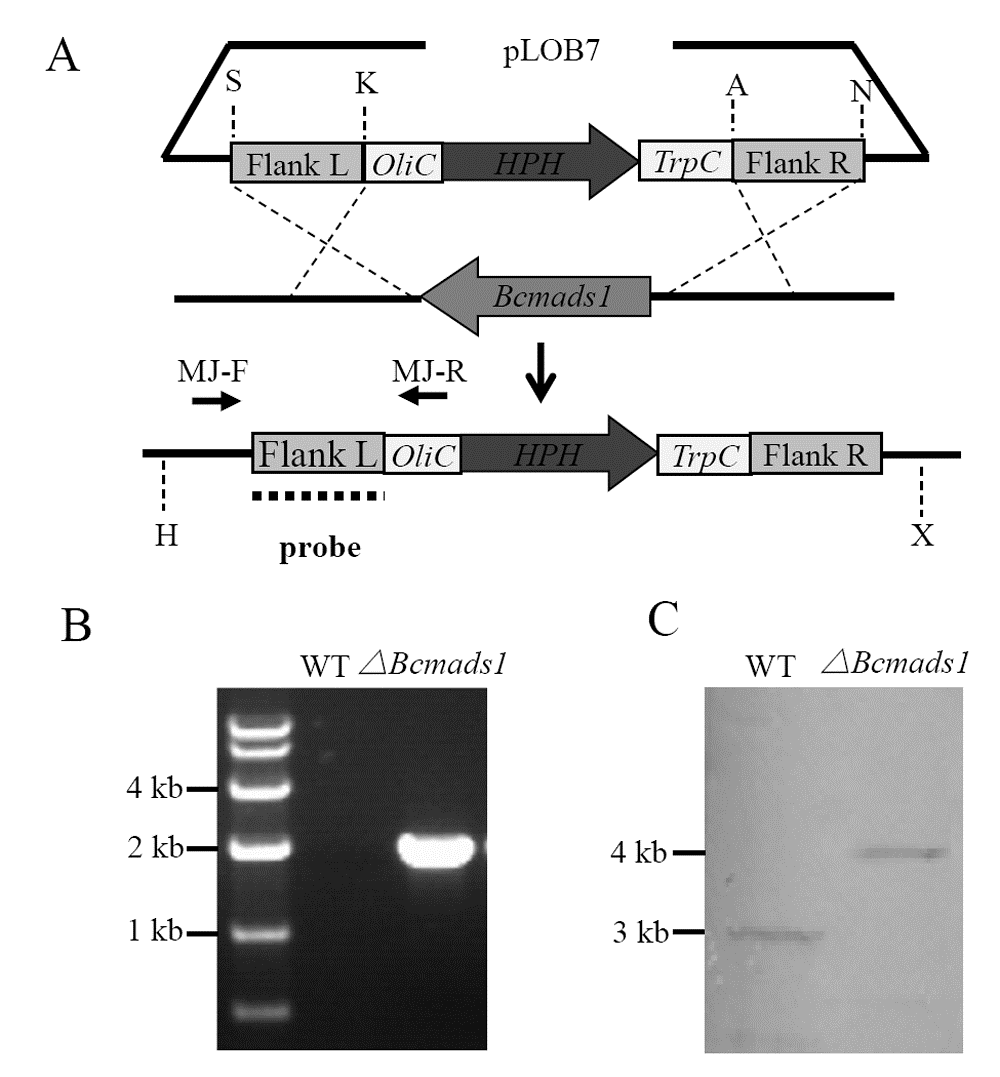


Fig. S2. Relative expression of *Bcmads* under light or dark conditions. Data presented represent the mean ± S.D. (n = 3). .


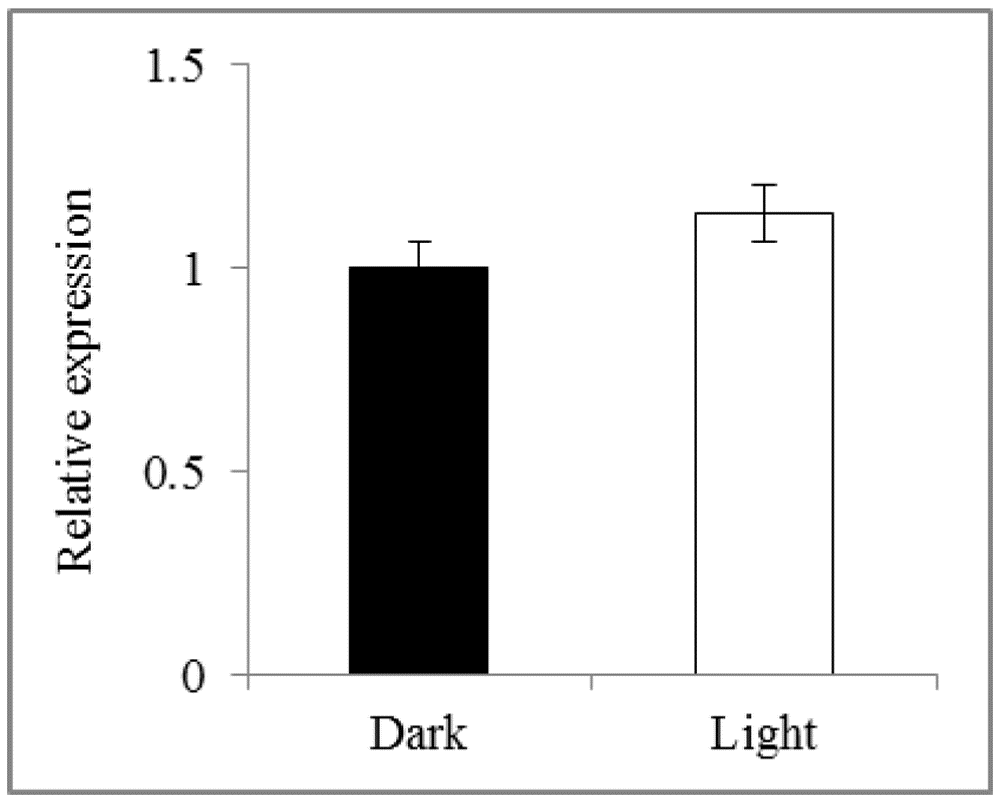


Fig. S3. Analysis of the proteome of wild-type and *△Bcmads* strains of *B. cinerea* using two-dimensional sodium dodecyl sulfate-polyacrylamide gel electrophoresis.


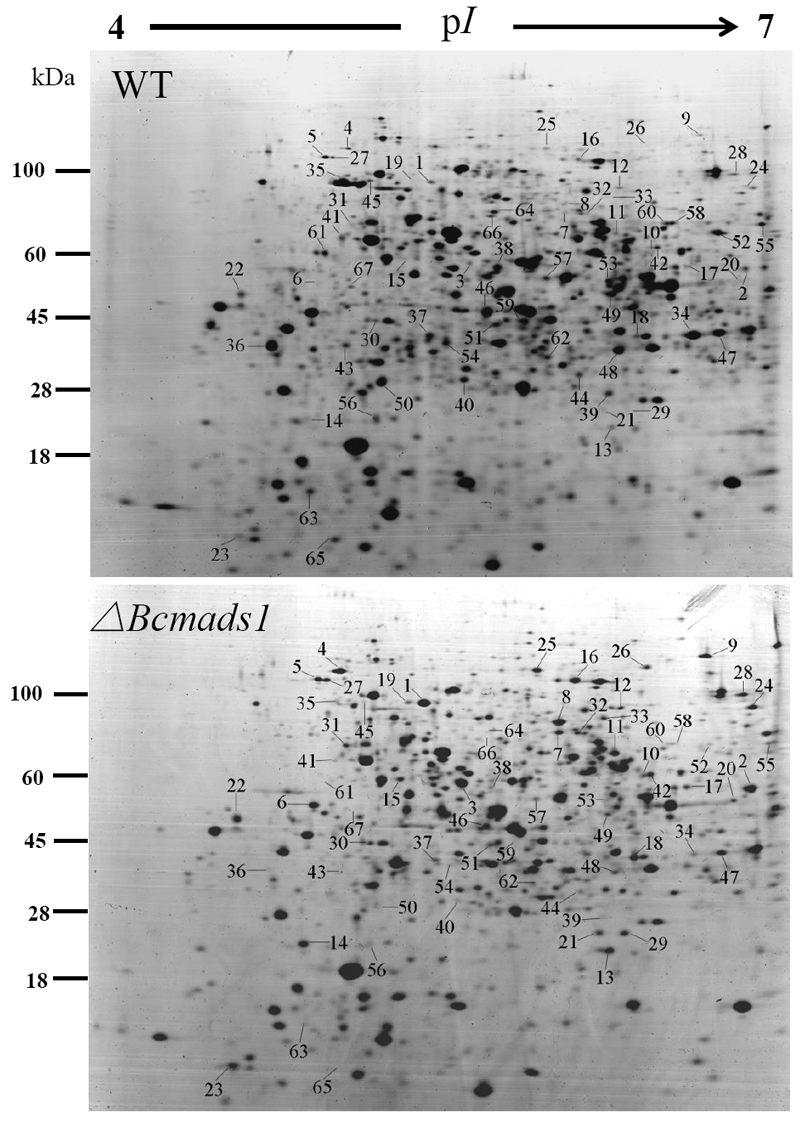


Fig. S4. Sclerotia formation in the complemented mutant strain *Bcmads-C* and its virulence on apple. A. The colony phenotype of the wild-type and complemented mutant strain *Bcmads-C* under light and dark conditions two weeks after inoculation. B. Comparison of the virulence of the wild-type and complemented mutant strain *Bcmads-C* strains of *B. cinerea*.


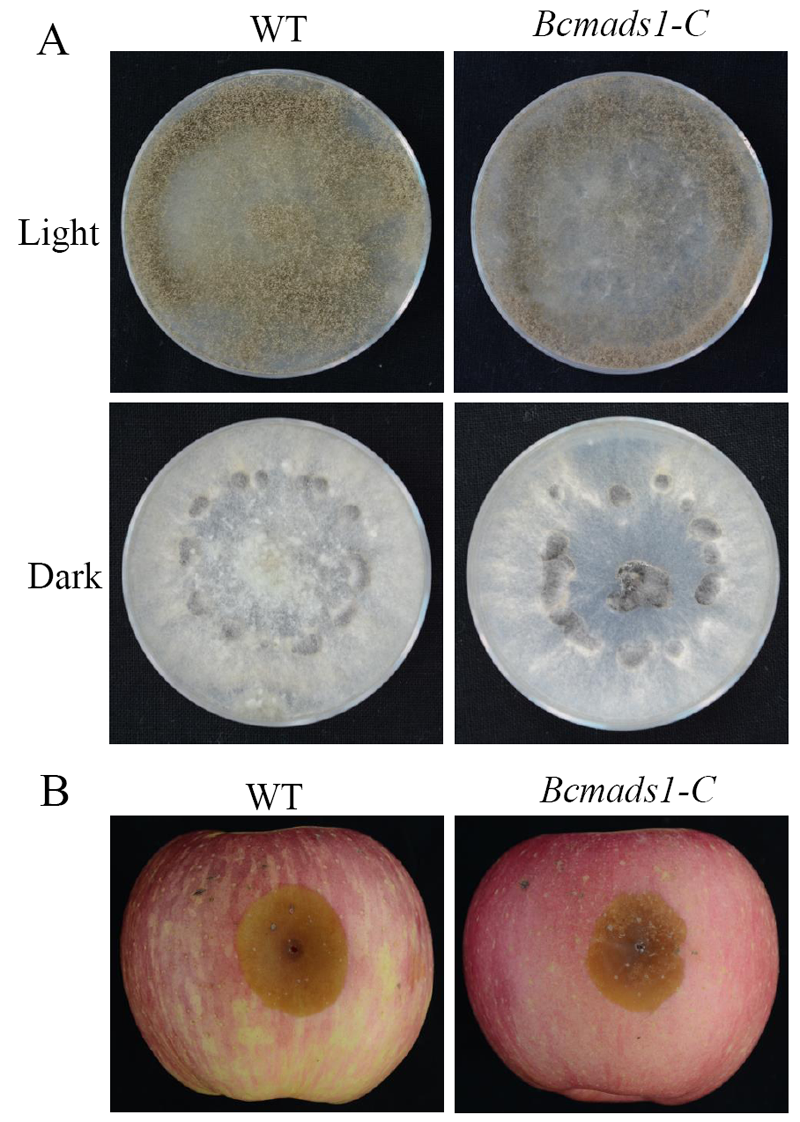


Fig. S5. Diagrammatic representation of the construction and generation of *Bcsec14* and *Bcsec31* mutants. A. Replacement strategy for deletion of *Bcsec14*. B. Detection of the mutant by flank-spanning PCR using the primer pairs 14J-F/14J-R. C. Replacement strategy for deletion of *Bcsec31*. D. Detection of the mutant by flank-spanning PCR using the primer pairs 31J-F/31J-R.


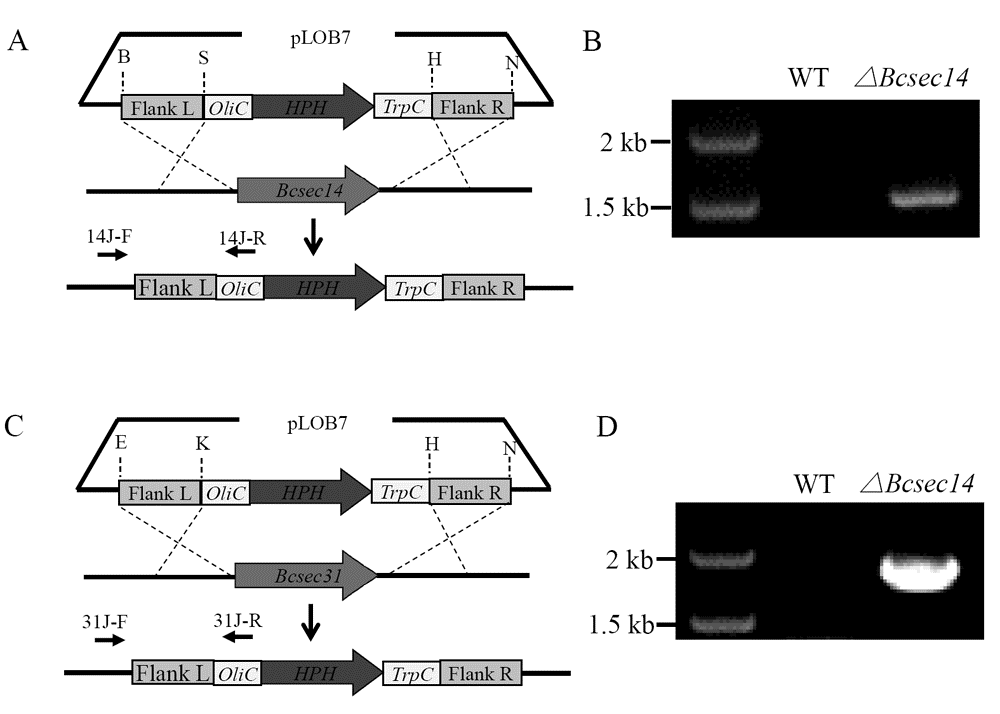


Fig. S5. Assay of conidial germination of WT and *△Bcsec14*/*△Bcsec31*. A. Germination rate of WT and *△Bcsec14*/*△Bcsec31* after incubating for 10 hours in PDB. Data represent the mean ± S.D. of three biological replicates. B. Image of the germinating conidia at 10 hour post-incubating.


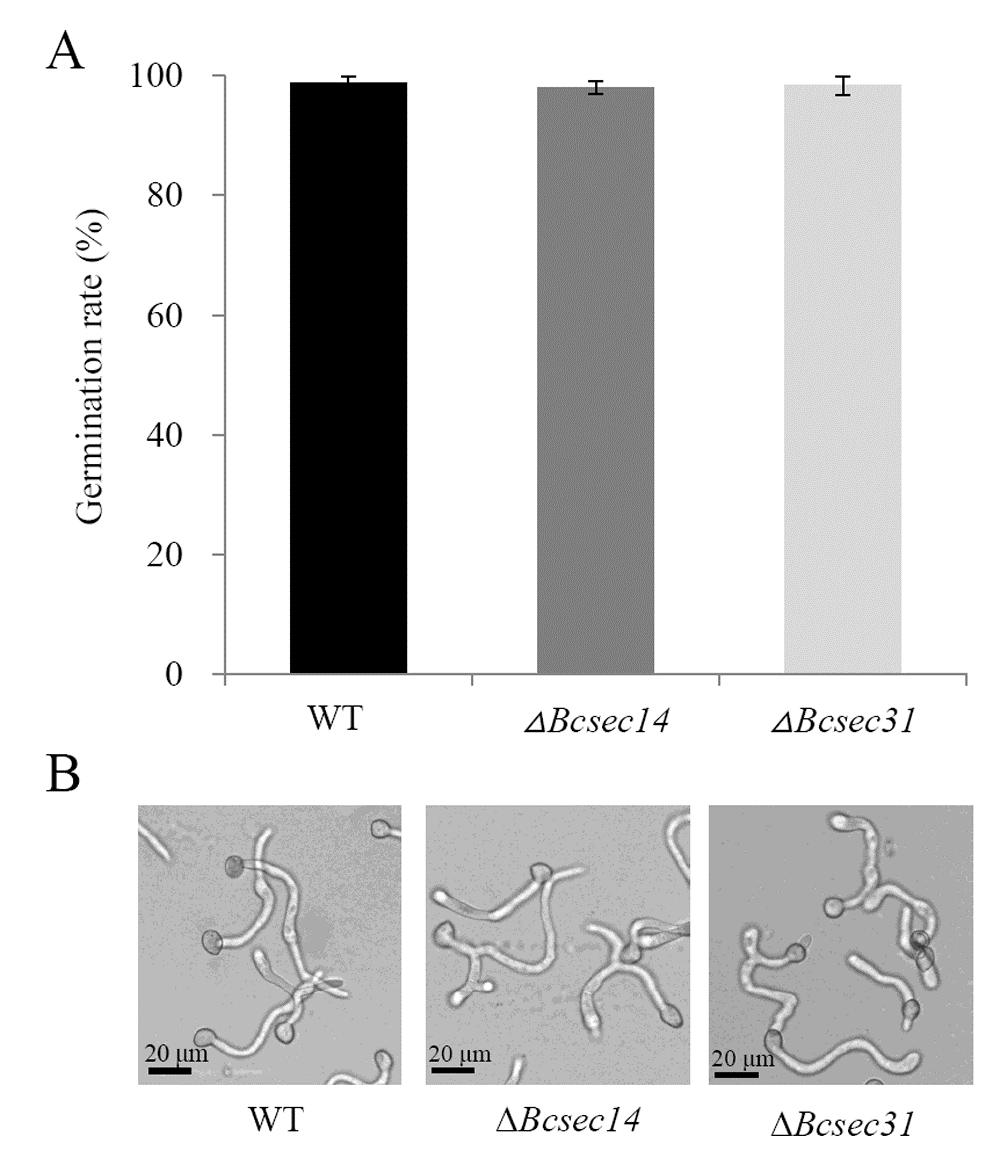


Table S1. Differentially abundant proteins identified by Q-TOF MS/MS.

| **Spot** | **Gene ID** | **Putative function** | **Mascot score**  **/ threshold** | **Fold**  **change** | **CArG**  **Box site** |
| --- | --- | --- | --- | --- | --- |
| **Heat shock protein and protein processing** | | | | |  |
| 1 | Bcin05g00180 | heat shock protein SSB | 1009/44 | 9.5↑ |  |
| 4 | Bcin10g00300 | ATP-dependent molecular chaperone HSC82 | 1040/45 | 11.1↑ |  |
| 5 | Bcin13g00960 | dnaK-type molecular chaperone BiP | 1002/44 | 2.4↑ |  |
| 9 | Bcin04g06570 | 5-methyltetrahydropteroyltriglutamate-homocysteine methyltransferase | 951/44 | 16.3↑ | -353 |
| 19 | Bcin05g00180 | heat shock protein SSB | 633/44 | 5.0↑ | -728, -242 |
| 27 | Bcin13g00960 | dnaK-type molecular chaperone BiP | 798/44 | 5.4↑ | -1080 |
| 32 | Bcin01g02670 | T-complex protein 1, beta subunit | 457/44 | 4.2↑ | -305 |
| 35 | Bcin10g00300 | ATP-dependent molecular chaperone HSC82 | 898/44 | 15.8↓ |  |
| 36 | Bcin03g06600 | heat shock protein 70 | 495/44 | 17.4↓ | -1802, -1090 |
| 45 | Bcin10g00300 | ATP-dependent molecular chaperone HSC82 | 414/74 | 3.9↓ | -1140 |
| 50 | Bcin05g00180 | heat shock protein SSB | 389/43 | 5.8↓ | -349 |
| 53 | Bcin03g06600 | heat shock protein 70 | 211/44 | 4.0↓ | -1802, -1090 |
| 56 | Bcin08g06700 | activator of heat shock protein 90 | 335/44 | 2.2↓ |  |
| **Redox reaction** | | | | |  |
| 14 | Bcin08g03610 | subunit IV of cytochrome c oxidase | 147/44 | 3.5↑ | -625 |
| 25 | Bcin02g05100 | copper methylamine oxidase precursor | 1180/44 | 5.6↑ | -1372 |
| 30 | Bcin03g07820 | NADH-ubiquinone oxidoreductase subunit | 270/44 | 2.1↑ | -292 |
| 38 | Bcin07g02610 | 6-phosphogluconate dehydrogenase | 330/44 | 4.7↓ | -640, -641 |
| 39 | Bcin01g08520 | mitochondrial peroxiredoxin PRX1 | 488/45 | 3.4↓ | -911 |
| 43 | Bcin07g02610 | 6-phosphogluconate dehydrogenase | 393/44 | 6.9↓ | -640, -641 |
| 44 | Bcin12g06430 | short chain dehydrogenase | 246/44 | 2.4↓ | -537, -306 |
| 46 | Bcin12g06430 | short chain dehydrogenase | 713/44 | 3.1↓ | -537, -306 |
| 47 | Bcin16g04800 | malate dehydrogenase | 796/44 | 2.9↓ | -1536, -1535  -1249 |
| 49 | Bcin10g05550 | cinnamyl alcohol dehydrogenase | 930/44 | 2.2↓ | -1206 |
| 51 | Bcin03g01560 | Aldo/keto reductase | 287/44 | 2.2↓ |  |
| 55 | Bcin04g05300 | glutathione reductase | 841/43 | 2.3↓ |  |
| 59 | Bcin13g05810 | aldehyde dehydrogenase | 489/44 | 6.3↓ |  |
| **Carbon and nitrogen metabolism** | | | | |  |
| 10 | Bcin11g03520 | S-adenosylmethionine synthetase | 964/44 | 5.0↑ | -534 |
| 18 | Bcin07g02370 | putative intracellular protease | 432/44 | 3.8↑ |  |
| 22 | Bcin02g08590 | vacuolar protease A precursor | 498/44 | 2.1↑ |  |
| 37 | Bcin07g06010 | thiazole biosynthetic enzyme | 357/44 | 4.0↓ |  |
| 42 | Bcin01g03640 | UTP-glucose-1-phosphate uridylyltransferase | 861/44 | 4.1↓ | -1676 |
| 48 | Bcin01g06600 | dihydrodipicolinate synthase family | 380/44 | 2.9↓ | -1929 |
| 52 | Bcin01g03640 | UTP-glucose-1-phosphate uridylyltransferase | 878/44 | 3.6↓ | -1676 |
| 62 | Bcin04g04490 | asparagine synthetase | 398/45 | 4.5↓ |  |
| 64 | Bcin01g09580 | bifunctional purine biosynthesis protein ADE16 | 190/44 | 4.5↓ | -1756 |
| 65 | Bcin08g02060 | ethanolamine utilization protein | 354/43 | 17.6↓ |  |
| **Energy metabolism** | | | | |  |
| 2 | Bcin02g02750 | citrate synthase, mitochondrial precursor | 702/44 | 2.9↑ |  |
| 7 | Bcin13g02430 | ATP-citrate synthase (ATP-citrate (pro-S-)-lyase) | 619/44 | 5.3↑ | -1328, -790 |
| 8 | Bcin02g06580 | pyruvate decarboxylase | 1219/43 | 9.1↑ | -174 |
| 15 | Bcin15g01380 | ATP-specific succinyl-CoA synthetase  beta subunit | 696/44 | 7.4↑ |  |
| 17 | Bcin07g04280 | isocitrate dehydrogenase subunit alpha | 649/44 | 4.1↑ | -1856 |
| 24 | Bcin09g01320 | isocitrate lyase | 840/44 | 12.4↑ | -1536 |
| 26 | Bcin02g05920 | hypothetical protein similar to aconitase | 214/44 | 14.4↑ |  |
| 28 | Bcin04g01780 | acetyl-CoA hydrolase | 549/45 | 3.7↑ |  |
| 29 | Bcin02g04170 | ATP synthase subunit 4, mitochondrial precursor | 526/44 | 5.6↑ |  |
| 40 | Bcin03g03360 | transaldolase | 69/44 | 3.1↓ |  |
| 54 | Bcin01g09950 | pyruvate carboxylase | 287/44 | 4.6↓ |  |
| 58 | Bcin15g04970 | glucose-6-phosphate isomerase | 362/44 | 2.2↓ |  |
| 60 | Bcin15g04970 | glucose-6-phosphate isomerase | 293/44 | 3.5↓ |  |
| **Cytoskeleton/Protein transport** | | | | |  |
| 3 | Bcin16g02020 | actin | 976/44 | 11.8↑ | -1696, -34 |
| 31 | Bcin01g08040 | tubulin beta chain | 938/44 | 2.7↑ | -595 |
| 57 | Bcin02g08570 | similar to sec14 cytosolic factor | 233/45 | 2.8↓ | -1537 |
| 66 | Bcin04g01050 | plastin-3 | 535/44 | 3.9↓ |  |
| 67 | Bcin14g04650 | putative transport protein sec31 | 695/44 | 3.1↓ |  |
| **Translation** | | | | |  |
| 6 | Bcin05g05820 | 40S ribosomal protein S0 | 544/44 | 9.3↑ |  |
| 33 | Bcin03g06250 | seryl-tRNA synthetase | 492/44 | 16.8↑ | -1256 |
| 34 | Bcin07g01540 | elongation factor 2 | 811/44 | 3.7↓ |  |
| **Others** | | | | |  |
| 16 | Bcin05g01260 | predicted protein | 481/44 | 4.9↑ | -1040 |
| 20 | Bcin03g06390 | mannose-1-phosphate guanyltransferase | 652/43 | 4.1↑ | -483 |
| 23 | Bcin03g00500 | allergen Asp f 15 precursor | 257/42 | 2.2↑ | -1968, -637 |
| 41 | Bcin16g03080 | vWFA superfamily | 280/44 | 3.3↓ | -1040 |
| 61 | Bcin05g01260 | predicted protein | 714/44 | 6.3↓ |  |
| 63 | Bcin13g02510 | protein wos2 | 127/44 | 3.2↓ |  |
